# Mediastinal Bronchogenic Cyst With Superior Vena Cava Syndrome: A Case Report

**DOI:** 10.7759/cureus.42040

**Published:** 2023-07-17

**Authors:** Annalee Mora, Amirali Ghavamrezaii, Hayder Abidali, Hector Caballero, Alisher Hamidullah, Nikolay Mitzov

**Affiliations:** 1 Internal Medicine, Oak Hill Hospital, Brooksville, USA

**Keywords:** duplication cyst, superior vena cava obstruction, mediastinal mass, superior vena cava syndrome, bronchogenic cyst

## Abstract

Bronchogenic cysts are rare in adults and often remain undetected until discovered incidentally on imaging or during a symptomatic investigation. The possibility of superior vena cava (SVC) compression due to a bronchogenic cyst arises in complex patient presentations. SVC syndrome poses several unique challenges due to the wide range of clinical symptoms and difficulty identifying the cause when symptoms first manifest. This case report examines a 39-year-old male who presented with symptoms resulting from SVC compression caused by a bronchogenic cyst, leading to SVC syndrome. We discuss the various imaging modalities used to assess the severity of the obstruction and the surgical interventions employed to alleviate the symptoms. A surgical intervention provides symptomatic relief and promises an excellent prognosis when performed without complications.

## Introduction

Bronchogenic cysts represent a rare congenital condition in adults, resulting from aberrations during embryonic respiratory system development [1,2]. These cysts can be classified as mediastinal, intrapulmonary, or ectopic, contingent on the timing of the developmental anomaly [1,3]. They typically manifest in the mediastinum, particularly the middle part. Although the early stages of the disease are usually asymptomatic, these cysts may grow over time, exerting pressure on surrounding tissues and organs, including the superior vena cava (SVC). This can lead to clinical symptoms of SVC syndrome, which can be life-threatening.

We report a unique case of a bronchogenic cyst in a middle-aged man who experienced sudden chest pain, dyspnea, dysphagia, and hoarseness. The patient was found to have a large bronchogenic cyst in the middle mediastinum compressing the SVC, a scenario typically treated through surgical resection. The initial radiographic diagnosis can be challenging due to variable presentation. Imaging studies, including chest X-ray (CXR), CT scan, and MRI, assist in identifying and characterizing these cysts. Histopathology following surgical resection provides a definitive diagnosis.

## Case presentation

A 39-year-old male with no significant medical history presented to the emergency department with sudden onset of dyspnea, dysphagia, hoarseness, and a sensation of halted blood flow to the brain when bending forward or lifting objects. His symptoms improved with head elevation. Over the previous week, he experienced intermittent chest pain and dyspnea during exertion, accompanied by headaches. A few years earlier, he reported brief, activity-associated episodes of dizziness. He denied experiencing fever, fatigue, anorexia, cough, palpitations, or weight loss and reported no history of chest trauma, smoking, alcohol, or illicit drug use.

His vital signs upon examination included a blood pressure of 133/89 mmHg, heart rate of 69 beats per minute, respiratory rate of 14, temperature of 36.9°C, and oxygen saturation of 99% on room air. He was awake, alert, and oriented, without acute distress. His head was normocephalic with no visual field defects. There were no signs of neck swelling or facial plethora. Lung auscultation was clear, and no heart murmurs were detected. His abdomen was soft, nontender, and free of organomegaly. No edema was noted in either the upper or lower extremities, and pulses were present.

**Figure 1:**
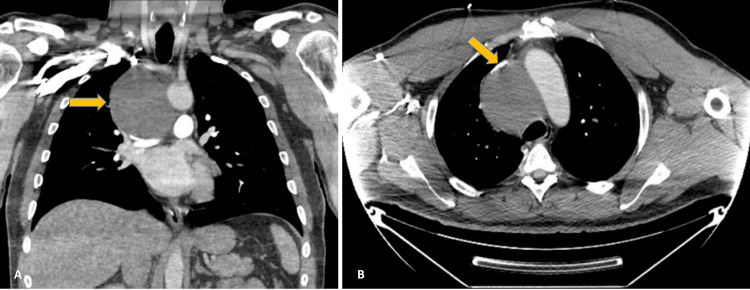
(A) CT scan of the chest with homogenous soft-tissue mass in the mediastinum. (B) Mass causing stenosis of the SVC with compression of the distal trachea and carina

The patient’s complete blood count, coagulation profile, troponin levels, and renal and liver function tests were within reference ranges. An electrocardiogram indicated a normal sinus rhythm. His CXR revealed an abnormal superior mediastinal contour, suggesting a mediastinal or pulmonary mass. A contrast-enhanced CT scan of the chest (Figure 1A) demonstrated a homogenous soft tissue density mass, measuring 7.3 x 8 x 8 cm in the mediastinum, raising concerns for neoplasm or lymphoma. The mass severely stenosed the SVC (Figure 1B), causing venous congestion and distal trachea and carina compression. CT of the soft tissue neck showed no abnormal findings.

**Figure 2:**
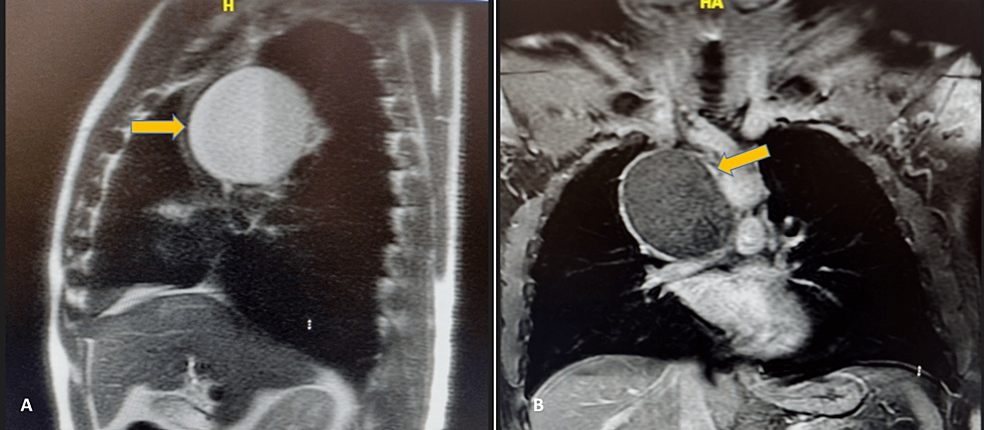
(A) Sagittal view of MRI of the chest. (B) Coronal view of MRI of the chest

An interventional radiologist initially planned to perform a tissue biopsy of the mediastinal mass. However, after consulting with the cardiothoracic surgery (CTS) team, the mass was suspected to be cystic rather than solid. Further evaluation with contrast-enhanced MRI of the chest (Figure 2A, 2B) confirmed an 8.7 x 7.6 x 7.9-cm unilocular, non-enhancing cystic mass in the middle mediastinum located in the lower right paratracheal region, with no septations or solid components. The mass caused significant lateral displacement and compression of the SVC.

**Figure 3:**
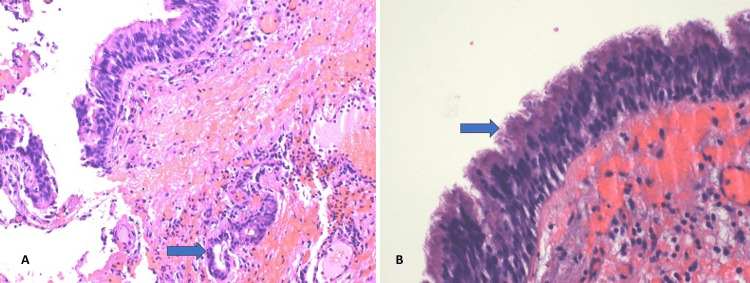
Hematoxylin and eosin stains of (A) the submucosal gland in 20x magnification. (B) Ciliated bronchial epithelium in 40x magnification

**Figure 4:**
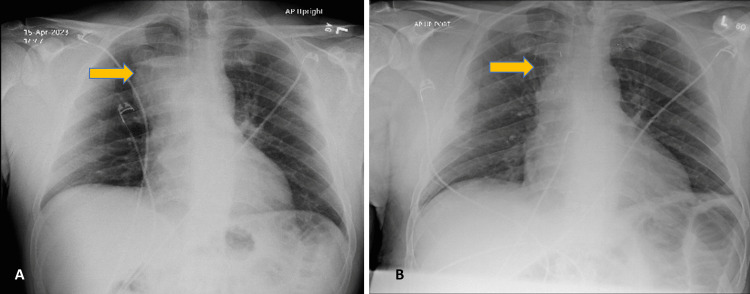
CXR before (A) and after (B) the surgical resection of the bronchogenic cyst

After discussions with the patient and his family, a cyst resection via right thoracotomy was performed. Gross and histopathological analysis of the submucosal glands (Figure 3A) and ciliated bronchial epithelium (Figure 3B) confirmed the benign nature of the bronchogenic cyst. The patient showed no immediate postoperative complications, and his symptoms dramatically improved. Follow-up CXR demonstrated no pneumothorax and no abnormal superior mediastinal contour. Comparative CXR images before and after the surgery (Figure 4) showed significant improvement. The patient was discharged 48 hours post-surgery, with a follow-up visit scheduled in the CTS clinic within one to two weeks.

## Discussion

Bronchogenic cysts, also known as duplication cysts, are congenital malformations that arise from abnormal budding of the embryological foregut during fetal development [1]. They typically present as solitary cysts within the mediastinum, although they can appear anywhere in the lungs [4]. The cystic mass in our patient was in the middle mediastinum, where 12% to 20% of mediastinal masses occur [5]. The cysts are lined histologically with respiratory epithelium and contain cartilage, glandular tissue, and smooth muscle [1,6]. Bronchogenic cysts can manifest at any age but are rare in adults. While 75% of patients remain asymptomatic [7] and cysts are usually detected incidentally on chest radiographs [8], significant growth can compress the surrounding structures, including the airways and blood vessels, such as the SVC. This can lead to symptomatic presentations.

Obstruction of the SVC can occur due to thrombosis, intrinsic stenosis, or extrinsic compression [9]. This can result in vascular congestion and backflow, leading to the clinical symptoms of SVC syndrome. The appearance and severity of these symptoms depend primarily on the mass’s location and size. Our patient’s cyst was large enough to cause symptoms like chest pain, dyspnea, headaches, hoarseness, dysphagia, and dizziness on forward bending, typically associated with SVC syndrome [9]. Substernal chest pain can result from an inflammatory process and irritation of the parietal pleura. Compression or irritation of the bronchi and esophagus by the cyst can cause dyspnea and dysphagia [10]. Patients may present with facial plethora and neck and face swelling as the cystic mass progresses due to edema [11]. While malignancy accounts for 70% of SVC syndrome cases [12], 40% arise from nonmalignant etiologies [11], and bronchogenic cysts account for 1% of these [1]. If not promptly recognized, SVC syndrome can be life-threatening, with patients potentially deteriorating rapidly.

Bronchogenic cysts are usually detected incidentally on CXRs in adulthood [13] or during evaluations for other clinical concerns, such as dyspnea and chest pain, as was the case with our patient. The cyst typically appears as a well-defined solitary spherical or oval mass with homogeneous opacity on a CXR [4]. However, the inability of a CXR to differentiate solid masses from fluid-filled ones often necessitates further evaluation with a chest CT scan. Contrast-enhanced tomography can accurately reveal the cyst’s size, position, and relationship to tracheobronchial and vascular structures [4,14]. Our patient’s initial CXR scan was inconclusive because cyst densities can vary, making differentiation from solid lesions challenging [15]. Consequently, we performed a chest MRI, a highly sensitive and specific method for diagnosing a cystic mass [7].

Managing SVC syndrome effectively and promptly necessitates a multidisciplinary team approach. Our priority was to prevent hemodynamic compromise and life-threatening cerebral and upper airway symptoms. Severe SVC obstruction or occlusion can lead to significant morbidity and mortality [12]. Elevating the patient’s bed helped decrease hydrostatic pressure in the neck and head. Treatment options for symptomatic mediastinal cysts range from less invasive procedures (e.g., percutaneous aspiration) to more invasive procedures such as thoracotomy, video-assisted thoracoscopy, or sternotomy [16]. Surgical resection is the standard treatment for growing symptomatic cysts because of the potential complications, symptom development, and cyst size increase [17]. The choice of surgical approach depends on the surgeon’s experience, the cyst’s location, and the patient’s general condition [17]. Although recurrence is rare following successful surgical resection, we emphasized the importance of long-term follow-up with our patient and his family in case of incomplete resection or intraoperative spillage of the cystic contents due to dense adhesion [18].

## Conclusions

Diagnosing the cause of SVC obstruction can be challenging without appropriate imaging modalities. Our case underscores the unique presentation of a bronchogenic cyst in the middle mediastinum and the possible complications of such a congenital malformation. In this instance, the cyst’s significant size and location led to SVC obstruction and the patient’s clinical symptoms. Whenever a lesion is discovered, a high index of suspicion for malignancy is crucial, necessitating a swift biopsy to rule out this possibility. The definitive diagnosis of a bronchogenic cyst is usually confirmed by histological examination. Timely and appropriate surgical intervention significantly relieved our patient’s symptoms. This case highlights the importance of a multidisciplinary approach to management, combined with imaging modalities and histological examination, to provide the best possible care for such a treatable lesion.

## References

[1] McAdams HP, Kirejczyk WM, Rosado-de-Christenson ML, Matsumoto S (2000). Bronchogenic cyst: imaging features with clinical and histopathologic correlation. Radiology.

[2] Cook J, Yulia A, Wallis C, Pandya PP (2020). Fetal lung lesions. Fetal medicine: basic science and clinical practice (third edition).

[3] Battal B, Bozlar U, Ors F, Nikolla S, Tasar M, Tayfun C (2008). Superior vena cava syndrome caused by bronchogenic cyst: report of a case. Internet J Pulm Med.

[4] Parkar N, Javidan-Nejad C, Bhalla S (2021). The mediastinum, including the pericardium. Grainger and Allison's diagnostic radiology (seventh edition).

[5] Hsu DS, Banks KC, Velotta JB (2022). Surgical approaches to mediastinal cysts: clinical practice review. Mediastinum.

[6] Fukada Y, Endo Y, Nakanowatari H, Kitagawa A, Tsuboi E, Irie Y (2020). Bronchogenic cyst of the interatrial septum. Fukushima J Med Sci.

[7] Tiwari MK, Yadav R, Mathur RM, Shrivastava CP (2010). Mediastinal bronchogenic cyst presenting with dysphagia and back pain. Lung India.

[8] Ayub II, Balakrishnan H, Arshad AM, Manimaran N, Thangaswamy D, Chockalingam C (2019). A 44-year-old man with nonproductive cough and sensation of heaviness over the upper chest. Chest.

[9] Ameli-Renani S, Belli AM, Chun JY, Morgan RA (2021). Superior vena cava syndrome. Grainger and Allison's diagnostic radiology (seventh edition).

[10] (2023). Foregut cysts of the mediastinum. https://thoracickey.com/foregut-cysts-of-the-mediastinum/.

[11] Nwanna-Nzewunwa OC, Lennon P, Gebhardt BR, Couper G, Robich MP (2022). Facial plethora and markedly elevated central venous pressure after mitral valve repair, maze, and left atrial appendage occlusion procedures. J Cardiothorac Vasc Anesth.

[12] Azizi AH, Shafi I, Shah N, Rosenfield K, Schainfeld R, Sista A, Bashir R (2020). Superior vena cava syndrome. JACC Cardiovasc Interv.

[13] Pułtorak R, Korlacki W, Pasierbek M, Grabowski A (2016). Thoracoscopic resection of a bronchogenic cyst in a 17-year-old girl. Kardiochir Torakochirurgia Pol.

[14] Durhan G, Ardali Duzgun S, Akpınar MG, Demirkazık F, Arıyürek OM (2021). Imaging of congenital lung diseases presenting in the adulthood: a pictorial review. Insights Imaging.

[15] Cardinale L, Ardissone F, Cataldi A, Gned D, Prato A, Solitro F, Fava C (2008). Bronchogenic cysts in the adult: diagnostic criteria derived from the correct use of standard radiography and computed tomography. Radiol Med.

[16] Alqassieh R, Al-Balas M, Al-Balas H (2020). Anesthetic and surgical considerations of giant pericardial cyst: case report and literature review. Ann Med Surg (Lond).

[17] Wang X, Li Y, Chen K, Yang F, Wang J (2020). Clinical characteristics and management of primary mediastinal cysts: a single-center experience. Thorac Cancer.

[18] Kozu Y, Suzuki K, Oh S, Matsunaga T, Tsushima Y, Takamochi K (2014). Single institutional experience with primary mediastinal cysts: clinicopathological study of 108 resected cases. Ann Thorac Cardiovasc Surg.

